# Arthroplasty Has Surpassed Surgical Fixation for Radial Head Fractures Among ABOS Oral Examination Candidates: A 19-Year Observational Study

**DOI:** 10.3390/jcm14176312

**Published:** 2025-09-06

**Authors:** Cole M. Patrick, Alexis B. Sandler, Kyle J. Klahs, John P. Scanaliato, Michael D. Baird, Nata Parnes

**Affiliations:** 1 Department of Orthopaedic Surgery and Rehabilitation, William Beaumont Army Medical Center, Fort Bliss, 18511 Highlander Medics St., El Paso, TX 79918, USA; 2 Department of Orthopaedics, Texas Tech University Health Sciences Center El Paso, El Paso, TX 79905, USA; 3 Department of Orthopaedics, Walter Reed National Military Medical Center, Bethesda, MD 20889, USA; 4 Department of Orthopaedic Surgery, Carthage Area Hospital, Carthage, NY 13619, USA; 5 Department of Orthopaedic Surgery, Claxton-Hepburn Medical Center, Ogdensburg, NY 13669, USA

**Keywords:** ABOS, fellowship, radial head fracture, radial head arthroplasty, open reduction internal fixation

## Abstract

**Background/Objectives**: Radial head arthroplasty (RHA) and open reduction and internal fixation (ORIF) have emerged as predominant methods of surgical management for radial head fractures. The objective of this study was to evaluate national trends in management of radial head fractures among ABOS oral examination candidates and to compare complication rates between RHA and ORIF. **Methods**: A search of the American Board of Orthopaedic Surgery (ABOS) oral examination database identified radial head fractures treated with RHA or ORIF between 2003 and 2021 in patients 18 years or older. **Results**: RHA cases increased significantly from 2003–2021 (*p* < 0.001). Patients undergoing RHA were older (52.4 years vs. 42.9 years, *p* < 0.001) and predominantly female (60.8% vs. 45.7%, *p* < 0.001). Medical and surgical complications within 60 days were higher after RHA (2.9% vs. 1.6%, *p* = 0.012; 24.9% vs. 20.4%, *p* = 0.001), most commonly stiffness (10.8% vs. 7.1%, *p* < 0.001), nerve injury (3.3% vs. 2.7%, *p* = 0.26), and implant failure (3.4% vs. 2.4%, *p* = 0.064). Non-union or delayed union (0.5% vs. 2.5%, *p* < 0.001) was significantly higher after ORIF, and fracture (1.1% vs. 0.3%, *p* = 0.008) was significantly higher after RHA. The highest proportion of RHA to ORIF was performed by surgeons with shoulder and elbow fellowship training (*p* < 0.001). **Conclusions**: Among ABOS Candidates, RHA volume surpassed ORIF for radial head fractures in 2010. Surgical complication rates for radial head fractures are high at 60 days follow-up for both procedures. RHA is associated with higher complication rates, especially stiffness; however, similar reoperation and readmission rates suggest that RHA may have been selected for more complex injuries.

## 1. Introduction

Radial head fractures are the most frequent elbow fracture pattern in the general adult population. They account for 75% of all elbow fractures and have an estimated incidence of 2.5 to 2.9 per 10,000 people annually [1,2,3]. The Mason Classification of radial head fractures, initially proposed in 1954, both guides treatment and classifies fracture patterns into four types: non-displaced or displaced <2 mm (type I); displaced ≥2 mm (type II); displaced and comminuted (type III); and fractures with concomitant elbow dislocation (type IV) [4,5,6].

As early as 1924, Speed noted that “unless the lesion is only a mere crack, there is no doubt that removal of the head is primarily indicated [7].” Over the last century, radial head excision, open reduction and internal fixation (ORIF), and radial head arthroplasty (RHA) have emerged as the dominant surgical management options; however, radial head excision has increasingly fallen out of favor. Radial head excision leads to high rates of elbow instability and radiographic osteoarthritis attributed to increased load on the ulnohumeral joint [8,9,10]. There seems to be consensus that, when possible, ORIF is preferred when stable fixation can be achieved; however, RHA is an acceptable alternative when reconstruction is not feasible due to severe fracture comminution or instability [11,12,13]. A series of biomechanical studies in the 1980s established RHA as a more favorable treatment for non-reconstructible radial head fractures. Conversely, the comparison of ORIF and RHA for reconstructible fractures in current literature yields conflicting conclusions and a lack of obvious procedural superiority, with evidence of improved short-term functional outcomes with RHA countered by increased risks of implant failure and higher revision rates [2,9]. Recent systematic reviews and database studies continue to show conflicting conclusions regarding outcomes between RHA and ORIF [14,15]. Importantly, prior work has focused on patient outcomes, whereas little is known about the adoption of procedures among early-career surgeons. Our study fills this gap by examining trends in surgical practice patterns among ABOS candidates and associated complication rates.

In the current study, we assessed trends in popularity and adverse events after RHA versus ORIF for radial head fractures over the last two decades among American Board of Orthopaedic Surgery (ABOS) Part II Oral Examination candidates at 60 days follow-up. Our hypotheses were that the use of RHA versus ORIF varies between surgeons with different subspecialty fellowship training and that shoulder and elbow fellowship-trained surgeons perform RHA for radial head fractures more frequently than surgeons from other subspecialties. We further sought to explore correlations between surgical complication rates among the pertinent subspecialties. Identifying trends in surgical procedures will guide understanding of the current state of radial head fracture management in surgeons new to practice and better characterize how these patterns have changed over time.

## 2. Materials and Methods

### 2.1. Data Source

The ABOS process for board certification in orthopaedic surgery involves two examinations: a written examination (Part I) that must be passed prior to an oral examination (Part II) that is taken after a minimum of 17 consecutive months of clinical practice. During the oral examination, candidates present cases from a 6-month window in which they are required to submit case logs documenting patient demographics, International Classification of Diseases (ICD)-9 or ICD-10 and Current Procedural Terminology (CPT) codes, follow-up periods, and whether they had medical or surgical complications for all cases performed. The database includes all cases submitted by candidates prior to the ultimate 12 case selection by ABOS for oral presentation. Despite the self-reported nature of data in the ABOS database, heightened scrutiny of cases as part of the exam establishes the database as an accurate reflection of practice among the ABOS candidates, many of whom are early in their careers. For this study, case log data sourced from the ABOS oral examination database were used to assess management of radial head fractures with RHA and ORIF. This study was exempt from Institutional Review Board approval given that database information is de-identified.

### 2.2. Database Query

The ABOS oral examination database was queried for all radial head fractures treated with RHA (CPT code 24666) or ORIF (CPT code 24665) [16]. The selected date range included 2003 through 2021, as prior to 2003, fellowship subspecialty training was not available for analysis. ICD-9 and ICD-10 codes were reviewed and ultimately only injury codes that pertained to radial head fractures were included in this analysis (813 for ICD-9 and S52.12 for ICD-10). All patients were at least 18 years of age.

### 2.3. Statistical Analysis

Proportions of radial head fractures treated with RHA versus ORIF were calculated and compared by year and geographic practice location. Patient demographics and adverse events were isolated and compared between RHA and ORIF. Proportions of fractures treated with RHA versus ORIF, odds ratios (OR) of treatment with RHA versus ORIF, and rates of surgical complications after RHA versus ORIF at 60 days follow-up were calculated and compared by type of fellowship training. Statistical analyses were performed using linear regression to assess trends over the 19-year study period as well as Student *t* tests to compare continuous data series with a normal distribution of variance. Chi-squared or Fisher’s exact tests were used to compare categorical variables between cohorts, with chi-squared tests used for cohorts with over five values and Fisher’s exact tests for cohorts with five or fewer values. Statistical significance was determined by an alpha value set to 0.05.

### 2.4. Summary of Methodology

Identify relevant ICD-9/10 codes for radial head fractures and CPT codes for ORIF and RHA → ABOS database query for 2003–2021 (*n* = 6120) → adult patients ≥ 18 years (*n* = 5070) → ICD/CPT code filtering → final sample size (*n* = 3509) → sub-analysis based on treatment with RHA (*n* = 1747) and ORIF (*n* = 1762).

## 3. Results

### 3.1. Procedural Frequency

**Figure 1:**
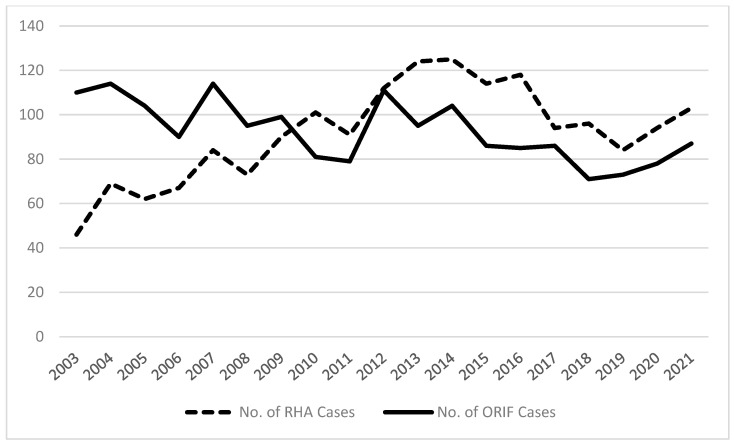
Chart demonstrating the trends in RHA and ORIF for Radial Head Fractures by American Board of Orthopedic Surgery Part II candidates from 2003–2021.

The proportions of radial head fractures treated with RHA versus ORIF (1747/1762) by ABOS Part II candidates has increased significantly between 2003 and 2021, (*p* < 0.001) (Table 1, Figure 1). Additionally, the number of RHA performed has increased steadily over this timeframe (*p* = 0.003), while the number of ORIF has declined (*p* < 0.001).

### 3.2. Demographics

Average patient age was significantly higher in patients undergoing RHA versus ORIF at 52.4 years and 42.9 years, respectively (*p* < 0.001) (Table 2). A greater proportion of patients undergoing RHA were female (60.8% vs. 45.7%, respectively; *p* < 0.001). Average follow-up times were not statistically different between groups (9.5 weeks for RHA, 8.9 weeks for ORIF; *p* = 0.083).

### 3.3. Adverse Events

Medical complications and surgical complications, self-reported by the ABOS candidates, at 60 days follow-up occurred at a significantly higher rate in patients after RHA versus ORIF (2.9% vs. 1.6%, *p* = 0.012; 24.9% vs. 20.4%, *p* = 0.001, respectively) (Table 2). The most common specified surgical complications included stiffness (10.8% after RHA vs. 7.1% after ORIF, *p* < 0.001), nerve injury (3.3% after RHA vs. 2.7% after ORIF, *p* = 0.26), and implant failure (3.4% after RHA vs. 2.4% after ORIF, *p* = 0.064) (Table 3). Non-union and delayed union were significantly higher after ORIF (0.5% after RHA vs. 2.5% after ORIF, *p* < 0.001), and fracture was significantly higher after RHA (1.1% after RHA vs. 0.3% after ORIF, *p* = 0.008). Rates of infection (1.4% after RHA vs. 0.7% after ORIF, *p* = 0.065), dislocation (1.0% after RHA vs. 0.6% after ORIF, *p* = 0.17), and wound healing (0.9% after RHA vs. 0.6% after ORIF, *p* = 0.42) were similar between the two procedures. Rates of reoperation and readmission did not vary significantly between RHA and ORIF (5.3% vs. 3.5%, *p* = 0.094; 2.9% vs. 1.7%, *p* = 0.10) (Table 2). Complication rate did not statistically change per year.

### 3.4. Geographic Practice Location

Proportions of RHA versus ORIF varied in different geographic regions (*p* = 0.002) (Table 4). Of the specified geographic locations, rates of RHA were highest in the Northeast (54.7%) while rates of ORIF were highest in the Northwest (55.5%).

### 3.5. Fellowship Training

The greatest proportion of RHA cases (64.5%) were performed by shoulder and elbow fellowship-trained surgeons, whereas the greatest proportion of ORIF cases (51.6%) were performed by sports medicine-trained surgeons (Table 5). Surgeons with shoulder and elbow fellowship training performed RHA at a higher rate than ORIF as compared to those with hand and upper extremity, sports medicine, and trauma fellowships (OR = 1.33, *p* < 0.001; OR = 1.33, *p* < 0.001; OR = 1.20, *p* = 0.002, respectively), whereas surgeons with trauma fellowships performed RHA at a higher rate than those with hand and upper extremity fellowships (OR = 1.11, *p* = 0.028) (Table 5). There were significant differences between surgical complications at 60 days follow-up after RHA and ORIF based on fellowship type (Table 6). As compared to surgeons with other fellowships, surgeons with hand and upper extremity fellowship training incurred higher rates of complications after RHA (31.3%, *p* < 0.001), and trauma trained surgeons had significantly higher rates of surgical complications after ORIF (24.6%, *p* = 0.004). The lowest rates of complications were seen in sports medicine fellowship-trained surgeons after RHA (22.8%) and hand fellowship-trained surgeons after ORIF (20.0%), although neither were significant (*p* = 0.035 vs. *p* = 0.72, respectively).

## 4. Discussion

This study sought to characterize existing differences between RHA and ORIF for the management of radial head fractures. Significant differences between the two procedures exist regarding procedural frequency, patient demographics, medical and surgical complications at 60 days follow-up, geographic practice location, and surgical fellowship training background. Between 2003 and 2021, there was an increase in the number of radial head fractures treated by ABOS oral candidates and the ratio of RHA to ORIF performed. Patients treated with RHA tend to be older and predominantly women. When compared to ORIF, RHA portends higher rates of medical and surgical complications despite similar rates of reoperation and readmission. Proportionally, shoulder and elbow fellowship-trained surgeons perform RHA for radial head fractures more frequently than other subspecialty-trained surgeons with similar rates of surgical complications. Hand fellowship-trained surgeons have a higher rate of surgical complications after RHA, and trauma fellowship-trained surgeons have higher rates of surgical complications after ORIF.

ORIF was consistently more common than RHA for radial head fractures prior to 2010. RHA for radial head fractures has steadily increased since 2003, matching rates of ORIF in 2010 and then surpassing it as the higher-volume procedure in the succeeding years. The increasing frequency of RHA is consistent with observed trends in the literature, which gave rise to a series of anatomic studies in the early 2000s and propelled diversification of RHA implant designs to better conform to patients’ anatomy [9,14]. Despite increasing rates of RHA, ORIF volumes are still described as approximately three times higher than RHA in studies prior to 2016 [17]. Our study is unique in demonstrating a steeper adoption rate and overall gross volume of RHA as compared to ORIF. ABOS oral candidates rapidly adopted RHA, and the persistence of this pattern over the last decade may indicate an overall shift in training and practice patterns favoring RHA over ORIF. Subsequently, the substantially higher volumes of RHA performed by ABOS candidates in the period after 2010 may have foreshadowed the overall shift towards RHA for radial head fractures by orthopaedic surgeons in general.

The high rate of surgical complications following RHA and ORIF aligns with the previously reported 10% to 75% complication rates [1,2,18,19]. However, the higher incidence of medical and surgical complications after RHA does not reflect previous studies showing no significant difference [2,11,14,15], or even lower complication rates after RHA [1,3,20,21]. Nevertheless, there is some evidence that shows better outcomes when ORIF can be achieved [22]. There are several possible explanations for these patterns. Regarding patient demographics, older patients undergoing RHA as opposed to ORIF may have factors that predispose them to medical and surgical complications. A recent large retrospective database study found that older patients and those with more comorbidities underwent RHA at a higher rate than ORIF [15]. Along the same lines, more challenging non-reconstructible fracture patterns may be treated with RHA over ORIF, predisposing these patients to worse outcomes. The short window for observing surgical complications in this study may also affect which ones were reported, as non-unions and hardware failure with ORIF may not be as readily observed as compared to dislocations or fractures with RHA. Finally, although there is limited evidence of higher complication rates after RHA, the procedure is nevertheless widely regarded as a technically challenging without clear learning curves discussed in existing literature [8,17]. With this in mind it is critical to emphasize that the observed complication rates in our study of ABOS oral examination candidates are similar to those reported by existing studies that were not limited to ABOS candidates [1,2,18,19].

At 60 days follow-up, the majority of complications occurred more frequently after RHA, with stiffness occurring at over triple the rate of nerve injury, infection, and implant failure. This high rate of stiffness after RHA was unexpected; a systematic review by Sun et al. [21], comparing RHA versus ORIF, demonstrated that ORIF heralded higher rates of bone nonunion/absorption and internal fixation failure, whereas rates of other complications including stiffness, nerve injury, infection, heterotopic ossification, and revision were comparable between the two procedures. This discrepancy may be explained in part by follow-up periods, given that stiffness is typically managed nonoperatively with physical therapy for the first 3 to 6 postoperative months, and the studies included in Sun et al. analysis had follow-up periods ranging from 1 to 3 years [21]. Nonunion/delayed union is primarily only relevant to ORIF as it is not biologically applicable after RHA. Nevertheless, the few instances of nonunion/delayed union after RHA in this study were secondary to concomitate procedures such as proximal ulna ORIF that led to nonunion and serves as a reporting artifact in the database used. For RHA, complications such as stiffness, implant failure, and fracture are more clinically meaningful.

Reoperation rates remained low after both RHA and ORIF, even with the surprisingly high rates of nerve injury and infection following both procedures. Even though stiffness is a key indication for reoperation after RHA, the notably higher rates of stiffness after RHA did not result in higher reoperation rates, which aligns with standard nonoperative management of stiffness for both procedures [9,23]. As previously discussed, inherent differences in fracture patterns or patient demographics may impact complication rates after RHA. Based on their assessment of reoperation rates in patients with radial head and neck fractures with and without elbow dislocation, Reinhardt et al. concluded that simple fractures may be better treated by ORIF, whereas RHA may be reserved for more severe injuries [24]. Although reoperation rates were similar between RHA and ORIF in the current study, an inclination towards RHA in the setting of more severe injuries due to concern for reoperation rates may also explain higher surgical complications after RHA.

Consistent with our hypothesis, shoulder and elbow fellowship-trained surgeons were performing RHA for radial head fractures at significantly higher rates than surgeons from other subspecialties. A similar trend was noted for other forms of upper extremity arthroplasty: ABOS oral examination candidates who are shoulder and elbow fellowship-trained performed total elbow arthroplasty for distal humerus fractures more frequently than other subspecialties in addition to total shoulder arthroplasty in general [25,26]. Interestingly, the highest rates of surgical complications after RHA were observed in hand and upper extremity fellowship-trained surgeons, which reached statistical significance. Trauma fellowship-trained surgeons had significantly higher rates of complications after ORIF as well. The variable rates of complications associated with different surgical subspecialties may reflect a selection bias that guides more complex cases towards certain fellowship-trained surgeons. For example, a study of complex scaphoid fractures treated by ABOS oral examination candidates postulated that hand fellowship-trained surgeons take on more complex fracture patterns as measured by the need for concomitant procedures [27]. Similarly, hand and trauma fellowship-trained surgeons may be referred complex radial head fractures that are more prone to complications than those treated by their colleagues or may gravitate towards more difficult cases after increased exposure to RHA and radial head ORIF in fellowship.

As with any database study, limitations in this investigation are rooted in limitations to the ABOS database. While data from the ABOS oral examination database are widely regarded as accurate reflections of cases, data are limited to cases recorded by candidates for the ABOS oral examination who are typically in practice for approximately 2 years, which limits the generalizability of our findings to surgeons very early in practice. These surgeries are being performed by surgeons early in their careers, so it may not be recommended to extrapolate complication rates to the general population. Additionally, data are constrained to characteristics reported in the ABOS database, thereby limiting comparison of patient age, comorbidities, and injury-specific details, such as fracture pattern, which may impact surgical management and the surgeon’s decision-making process. Time from injury and fracture complexity is not measured, with Mason I-IV fractures indistinguishable under the umbrella of the same ICD-10 code. The lack of injury- and implant-specific characteristics further introduces additional variability given the potential for heterogeneous levels of reconstructability seen in fractures included in this study, as well as the substantial evolution of RHA implants over the last several decades. Additionally, follow-up periods from the database were limited to approximately 60 days of follow-up on average, limiting outcome comparisons for complications and reoperations to this time window. There is also the opportunity for selection bias, as some candidate surgeons may avoid more complex cases by referring them to a colleague. Because this study examines surgeon-reported case logs rather than patient registry outcomes, conclusions should be interpreted as reflecting early-career surgeon practice patterns rather than long-term patient results. Decisions may be influenced by factors beyond clinical evidence, including training background, perceived implant reliability, and evolving surgical trends.

## 5. Conclusions

Among ABOS Candidates, RHA volume surpassed ORIF for radial head fractures in 2010. Patients should be counselled that surgical complication rates for RHF are high at 60 days follow-up for both procedures. Fellowship training may have an influence in treatment modality, with shoulder and elbow surgeons more likely to treat radial head fractures with RHA.

## Figures and Tables

**Table 1:** Trend of ABOS Oral Examination Candidates Performing RHA and ORIF for Radial Head Fractures by Year.

Exam Year	No. of Candidates	RHA Cases (%)	ORIF Cases (%)
2003	613	46 (29.5%)	110 (70.5%)
2004	698	69 (37.7%)	114 (62.3%)
2005	687	62 (37.3%)	104 (62.7%)
2006	655	67 (42.7%)	90 (57.3%)
2007	662	84 (42.4%)	114 (57.6%)
2008	664	73 (43.5%)	95 (56.5%)
2009	663	90 (47.6%)	99 (52.4%)
2010	680	101 (55.5%)	81 (44.5%)
2011	662	91 (53.5%)	79 (46.5%)
2012	722	112 (50.2%)	111 (49.8%)
2013	689	124 (56.6%)	95 (43.4%)
2014	770	125 (54.6%)	104 (45.4%)
2015	746	114 (57.0%)	86 (43.0%)
2016	729	118 (58.1%)	85 (41.9%)
2017	743	94 (52.2%)	86 (47.8%)
2018	752	96 (57.5%)	71 (42.5%)
2019	789	84 (53.5%)	73 (46.5%)
2020	767	94 (54.7%)	78 (45.3%)
2021	739	103 (54.2%)	87 (45.8%)
Total	13,430	1747	1762
*p* value	<0.001 ^a^	0.003 ^a^ (<0.001 ^a^)	<0.001 ^a^ (<0.001 ^a^)

Abbreviations: ABOS, American Board of Orthopedic Surgery; ORIF, open reduction internal fixation; RHA, radial head arthroplasty. ^a^ Statistically significant.

**Table 2:** Comparison of RHA and ORIF Case Frequency, Patient Characteristics, and Complications for Radial Head Fractures.

	Patient Age ^a^ (Years)	Female Patients	Follow Up (Weeks)	Medical Complications	Surgical Complications	Reoperation ^b^	Readmission ^b^
RHA	52.4	60.8%	9.5	51/1747 (2.9%)	435/1747 (24.9%)	50/952 (5.3%)	28/952 (2.9%)
ORIF	42.9	45.7%	8.9	29/1762 (1.6%)	359/1762 (20.4%)	27/765 (3.5%)	13/765 (1.7%)
*p*	<0.001 ^c^	<0.001 ^c^	0.083	0.012 ^c^	0.001 ^c^	0.094	0.010

Abbreviations: ORIF, open reduction internal fixation; RHA, radial head arthroplasty. ^a^ Age ≥ 18 years. ^b^ Collected from 2013–2021. ^c^ Statistically significant.

**Table 3:** Surgical Complications.

Type	Total	RHA Complications (%) (*n* = 1747 Cases)	ORIF Complications (%) (*n* = 1762 Cases)	*p*
Stiffness/arthrofibrosis	313	188 (10.8%)	125 (7.1%)	<0.001 ^a^
Other	226	119 (6.8%)	107 (6.1%)	0.37
Nerve palsy/Injury	105	58 (3.3%)	47 (2.7%)	0.26
Implant failure/malfunction	102	60 (3.4%)	42 (2.4%)	0.064
Non-union/delayed union	52	8 (0.5%)	44 (2.5%)	<0.001 ^a^
Infection	37	24 (1.4%)	13 (0.7%)	0.065
Loss of reduction	36	18 (1.0%)	18 (1.0%)	0.98
Dislocation	27	17 (1.0%)	10 (0.6%)	0.17
Wound healing	26	15 (0.9%)	11 (0.6%)	0.42
Bone fracture	25	19 (1.1%)	6 (0.3%)	0.008 ^a^
Pain	11	6 (0.3%)	5 (0.3%)	0.77

Abbreviations: ORIF, open reduction internal fixation; RHA, radial head arthroplasty. ^a^ Statistically significant.

**Table 4:** RHA and ORIF Cases for Radial Head Fractures Performed by ABOS Oral Examination Candidates by Geographic Practice Location.

	Midwest	Northeast	Northwest	South	Southeast	Southwest	Other
RHA	337 (48.3%)	400 (54.7%)	141 (44.5%)	292 (49.3%)	277 (52.1%)	295 (47.9%)	5 (21.7%)
ORIF	361 (51.7%)	331 (45.3%)	176 (55.5%)	300 (50.7%)	255 (47.9%)	321 (52.1%)	18 (78.3%)
Total	698	731	317	591	532	616	23

Abbreviations: ABOS, American Board of Orthopedic Surgery; ORIF, open reduction internal fixation; RHA, radial head arthroplasty.

**Table 5:** RHA and ORIF Cases by Type of Fellowship Training for ABOS Oral Examination Candidates.

Type	Hand and Upper Extremity	Shoulder and Elbow	Sports Medicine	Trauma
RHA and ORIF performed by type of fellowship training				
RHA	688 (48.6%)	187 (64.5%)	311 (48.4%)	363 (53.7%)
ORIF	729 (51.4%)	103 (35.5%)	332 (51.6%)	313 (46.3%)
Total	1417	290	643	676
Ratio of RHA to ORIF by type of fellowship training				
Hand and upper extremity		0.75 (*p* < 0.001 ^a^)	1.00 (*p* = 0.94)	0.90 (*p* = 0.028 ^a^)
Shoulder and elbow	1.33 (*p* < 0.001 ^a^)		1.33 (*p* < 0.001 ^a^)	1.20 (*p* = 0.002 ^a^)
Sports medicine	1.00 (*p* = 0.94)	0.75 (*p* < 0.001 ^a^)		0.90 (*p* = 0.053)
Trauma	1.11 (*p* = 0.028 ^a^)	0.83 (*p* = 0.002 ^a^)	1.11 (*p* = 0.053)	

Abbreviations: ABOS, American Board of Orthopedic Surgery; ORIF, open reduction internal fixation; RHA, radial head arthroplasty. ^a^ Statistically significant.

**Table 6:** Surgical Complication Breakdown by Type of Fellowship Training for ABOS Oral Examination Candidates.

	RHA	RHA Surgical Complications	*p*	ORIF	ORIF Surgical Complications	*p*
Hand and upper extremity	688	215 (31.3%)	<0.001 ^a^	729	146 (20.0%)	0.72
Shoulder and elbow	187	46 (24.6%)	0.92	103	28 (27.2%)	0.080
Sports medicine	311	71 (22.8%)	0.35	332	68 (20.5%)	0.98
Trauma	363	88 (24.2%)	0.74	313	77 (24.6%)	0.044 ^a^

Abbreviations: ABOS, American Board of Orthopedic Surgery; ORIF, open reduction internal fixation; RHA, radial head arthroplasty. ^a^ Statistically significant.

## Data Availability

Data for similar research studies can be obtained through the American Board of Orthopaedic Surgery (ABOS) oral examination database at https://www.abos.org/certification/. The data in this study was initially accessed on 6 March 2022.

## Abbreviations

The following abbreviations are used in this manuscript:

RHA	Radial head arthroplasty
ORIF	Open reduction and internal fixation
ABOS	American Board of Orthopaedic Surgery
ICD	International classification of diseases
CPT	Current procedural terminology
